# Putative human sperm Interactome: a networks study

**DOI:** 10.1186/s12918-018-0578-6

**Published:** 2018-04-11

**Authors:** Alessandra Ordinelli, Nicola Bernabò, Massimiliano Orsini, Mauro Mattioli, Barbara Barboni

**Affiliations:** 1 Faculty of Bioscience and Technology for Food, Agriculture and Environment, University of Teramo, Teramo, Italy; 2 Istituto Zooprofilattico Sperimentale “G. Caporale”, Teramo, Italy

**Keywords:** Spermatozoa, Interactome, Biological networks, Scale free, Small world, Male infertility, Systems biology

## Abstract

**Background:**

For over sixty years, it has been known that mammalian spermatozoa immediately after ejaculation are virtually infertile. They became able to fertilize only after they reside for long time (hours to days) within female genital tract where they complete their functional maturation, the capacitation. This process is finely regulated by the interaction with the female environment and involves, in spermatozoa, a myriad of molecules as messengers and target of signals. Since, to date, a model able to represent the molecular interaction that characterize sperm physiology does not exist, we realized the Human Sperm Interactme Network3.0 (HSIN3.0) and its main component (HSNI3.0_MC), starting from the pathway active in male germ cells.

**Results:**

HSIN3.0 and HSIN3.0_MC are scale free networks, adherent to the Barabasi-Albert model, and are characterised by an ultra-small world topology. We found that they are resistant to random attacks and that are designed to respond quickly and specifically to external inputs. In addition, it has been possible to identify the most connected nodes (the hubs) and the bottlenecks nodes. This result allowed us to explore the control mechanisms active in driving sperm biochemical machinery and to verify the different levels of controls: party vs. date hubs and hubs vs. bottlenecks, thanks the availability of data from KO mice. Finally, we found that several key nodes represent molecules specifically involved in function that are thought to be not present or not active in sperm cells, such as control of cell cycle, proteins synthesis, nuclear trafficking, and immune response, thus potentially open new perspectives on the study of sperm biology.

**Conclusions:**

For the first time we present a network representing putative human sperm interactome. This result gives very intriguing biological information and could contribute to the knowledge of spermatozoa, either in physiological or pathological conditions.

**Electronic supplementary material:**

The online version of this article (10.1186/s12918-018-0578-6) contains supplementary material, which is available to authorized users.

## Background

Mammalian spermatozoa are known to acquire their fertilizing ability only after a long and complex series of biochemical modification that recognises different steps. It starts within the testis, with spermatogenesis, and continues in epididymis, where spermatozoa undergo a series of important chemical modifications, and then it culminates with a process, called capacitation, that occurs within the female genital tract. Capacitation was discovered in early fifties, of the twentieth century independently by Austin [1, 2] and Chang [3] and in next sixty-five years, it has attracted the attention of groups of researchers from around the world. Nowadays we know several molecular events involved in spermatozoa physiology and in signal transduction pathways. It is clear that peculiar biological features characterize male gametes: virtually they are transcriptionally silent, their cytosol is absent, and they are the only cellular typology that exert its function in an organism other than that produced them. Consequently, they adapted their biochemical machinery to these conditions and evolved a complex control strategy. Their function is finely modulated by an uninterrupted dialogue with the surrounding environment, constituted by the cells and the fluids of female genital tract. By this way, they are able to physiologically responding to physical and chemical signals that arise from the neuro-endocrine activity of female. It has been found that sperm membrane and cytoskeleton play a key role in this process. Indeed, both of them are highly dynamical structures and undergo a deep reorganization during post-ejaculatory life of male gametes. The plasma membrane (PM) and the outer acrosome membrane (OAM) lose their asymmetry and become more fluid and isotropic, thus acquiring the ability to fuse (fusogenicity). The actin, first, polymerizes forming a network that separates PM and OAM, then it depolymerizes, allowing their contact and favoring the start of acrosome reaction (AR). All these events are caused and cause important modifications in the concentration of messengers, such as intracellular calcium concentration, [Ca^2+^]_i_; intracellular pH (pH_i_); cAMP, nitric oxide (NO), and intracellular concentration of bicarbonate ([HCO_3_^−^]_i_). Consequently, several target (kinases, phosphatases, lipases, cyclases, etc.) are activated originating a specific and regulated metabolic response [4–6].

Until a few years ago, the experimental works of researchers approaching the study of these events were limited for technical reasons. More recently, the adoption of high-througput technologies allowed obtaining a myriad of data. This had great positive consequences, as the rapid increase in the number of molecules and molecular event studied and the great amount of functional data obtained. In particular, the studies carried out by using proteomic approaches are giving very interesting results and offer important suggestion of new phenomena to be investigated [7, 8]. On the other side, unfortunately, we still lack of the ability to take inference from the data, in part due to the unavailability of models designed to take into account the biological complexity of these events. Indeed, the data per se are useless without the possibility to aggregate and interpreter them. The proof is that in a large number of patients with fertility problems and undergoing assisted reproductive technology (ART), it is impossible to reach an etiological diagnosis [9–11].

To overcome this limitation, here, we explored the possibility to apply a computational modelling approach, based on networks theory, to the definition and the study of spermatozoa interactome. Briefly, we used the data from published proteomic studies on pathways expressed in spermatozoa, to build a model in which the molecules are represented as nodes and the interactions among them as links, thus originating a network. We had already used this approach for studying specific events of sperm biology, such as the epididymal maturation [12], the capacitation [13–15], the cytoskeleton dynamics [16], or the sperm-egg binding and recognition [17]. Here, we propose a more global view of mature spermatozoa physiology, and the study of network architecture, the so-called topology, has allowed us to infer important biological features and to lay the foundations for further studies.

## Results

### Human spermatozoa Interactiome network (HSIN)

**Fig. 1:**
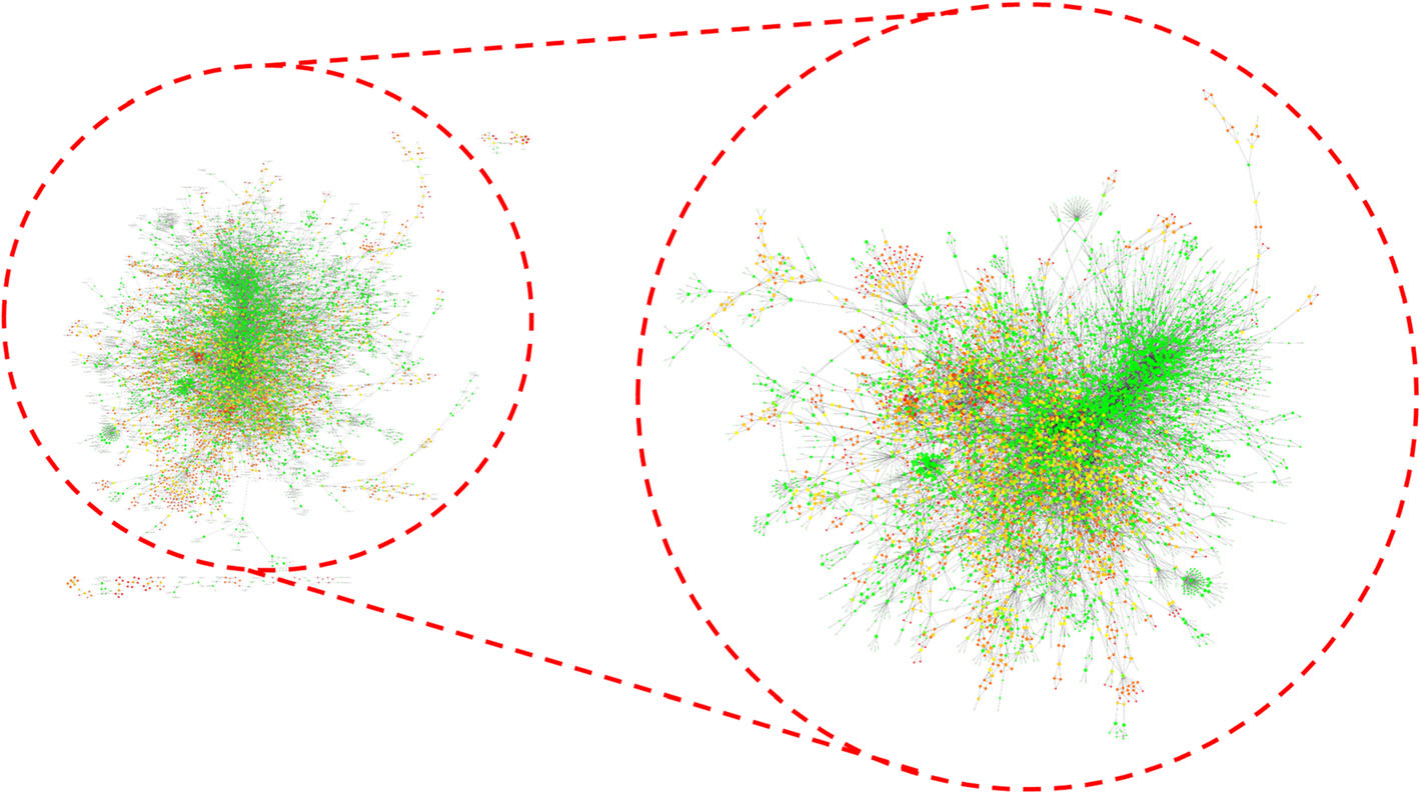
HSIN3.0 and HSIN.0_MC. The networks were spatially represented using the Cytoscape Prefuse Force Directed Layout. This program “is based on a” force-directed “paradigm. Network nodes are treated like physical objects that repel each other, such as electrons. The connections between nodes are treated like metal springs attached to the pair of nodes. These springs repel or attract their end points according to Functional in force function. The layout algorithm sets the positions of the nodes in a way that Minimizes the sum of forces in the network” (Cytoscape 3.4.0 User Manual http://manual.cytoscape.org/en/stable/index.html). The node diameter is directly proportional to the degree node and the node color gradient was dependent from the clustering coefficient, from red (higher) to green (lower)

As result of our data collection and refinement activity, we obtained different networks: HISN1.0 composed by 225 connected components (data not shown), HSIN2.0 composed by 104 connected components (data not shown), and HSIN 3.0 composed by 39 connected components (whose topological parameters are represented in Table 1). The final step of our work was the realization of HSIN3.0, composed by 25 connected components, 7891 nodes and 14,712 links, which represent the most complete evolution of HSIN. Since the larger connected component has 7758 nodes, thus representing 98.3% of HSIN 3.0, all the further analysis were carried out on this network, called HSIN 3.0 Main Component, HSIN3.0_MC (Table 1 and Table 2; Fig. 1).

**Table 1:** main topological parameter computed on HSI 1.0, HSI 2.0, HSI 3.0, MC_HSI 3.0 networks

Parameter	HSI 3.0	MC_HSI 3.0
Number of nodes	7892	7758
Number of edges	14,712	14,534
Clustering coefficient	0.073	0.070
Connected components	25	1
Network diameter	37	37
Shortest paths	27,139,995 (43%)	27,139,415 (45%)
Characteristic path length	10.320	10.321
Avg. number of neighbours	3.727	3.746

**Table 2:** Node degree distribution and correlation of node degree with clustering coefficient in MC_HSI 3.0

Node degree distribution	In-degree	Out-degree
- γ	−1.850	−1.764
R	0.985	0.963
R^2^	0.781	0.814
Clustering coefficient vs. node degree		
- γ	−1.628	
r	0.223	
R^2^	0.620

### Hubs: identification and subpopulations

**Fig. 2:**
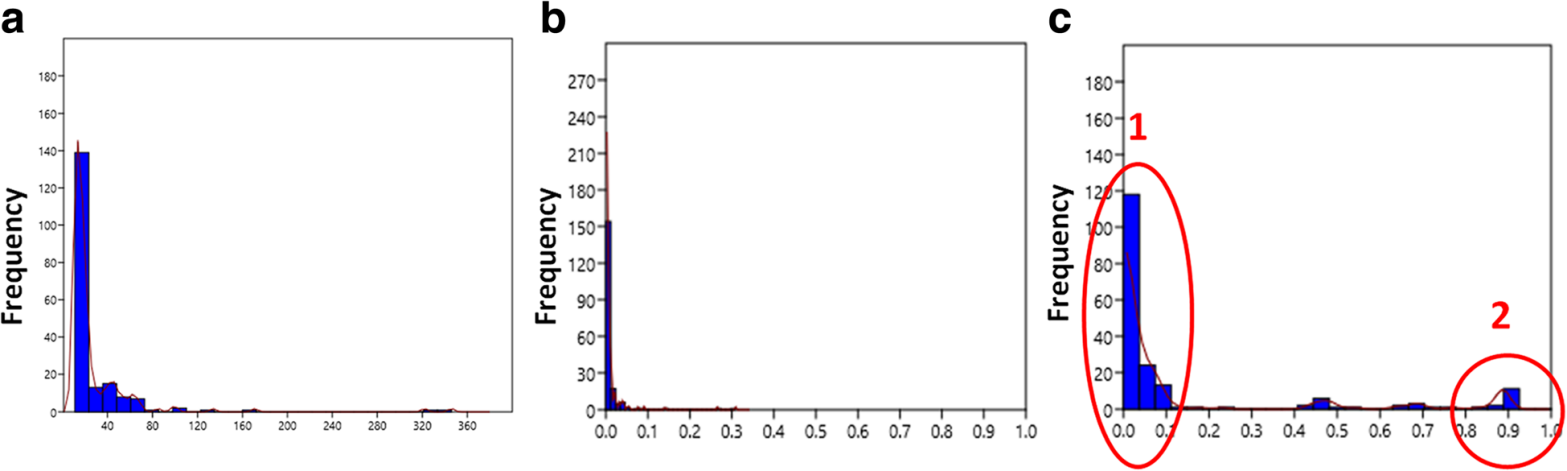
Graphs showing the result of KDE on main topological parameters of HSIN3.0_MC. **a** KDE analysis of node degree values. **b **KDE analysis of betweenness centrality. **c** KDE analysis of clustering coefficient

We found 190 hubs in HSIN3.0_MC (for hubs list and topological parameters see Additional file 1). Interestingly the KDE analysis we carried out allowed to identifying the presence of hubs subpopulations when analysing the clustering coefficient, as it is evident from the data depicted in Fig. 2.

### Identification of bottleneck nodes

The analysis of HSIN3.0_MC for bottleneck carried out with cytoHubba allowed to identify the nodes characterized by a higher score (see Additional file 2).

### Effect of the deletion of gene relative to protein listed in HSIN3.0_MC in KO mice on male fertility

We found that the genes coding for about 11% (248 on 2130) of the proteins present in HSIN3.0_MC have been studied in terms of fertility on mice KO models. More in detail, we investigated four different KO phenotypes: “male infertile”, “male hypofertile”, “male fertility affected”, and “male fertility unaffected/normal phenotype”. Interestingly, as it shown in Table 3, the hubs are more studied than the non-hubs genes (the data available are 35.6% for hubs and 19.6% for non-hubs), and the percentage of protein whose genes are related with fertility did not differ in hubs in comparison with non-hubs (*p* = 0.21991, Chi^2^ test). On the contrary, the deletion of genes codifying for the bottleneck protein in 100% of cases caused infertility.

**Table 3:** phenotypical effects of the deletion of gene relative to protein listed in HSIN3.0_MC in KO mice on male fertility

	Hubs N (%)	Non-hubs N (%)
Male infertility	5 (23.8)	96 (27.8)
Male hypofertility	3 (14.3)	60 (17.4)
Male fertility affected	3 (14.3)	95 (27.5)
Male fertility unaffected/normal phenotype	10 (47.6)	94 (27.2)
Total	21 (100)	345 (100)

Male infertility: male KO is infertile; Male hypofertility: male KO has reduced fertility in comparison with wild type; Male fertility affected: KO mice have a damage in reproductive function with unclear or unknown effects on male fertility; Male fertility unaffected/normal phenotype: the KO mice have the same fertility of wild type

## Discussion

In this work, we realized the networks representing the putative Human Spermatozoa Interactome (HSIN3.0 and HSIN3.0_MC), starting from published proteomic data. Once obtained the network model, we assessed its topology to infer important biological information. As first, it is evident that HSIN3.0 and HSIN3.0_MC are characterised by a specific topology: they are scale free networks in keeping with the Barabasi-Albert (BA) model. They are characterised by a wide heterogeneity of node degree of nodes. In particular, in BA networks a low number of highly connected nodes (the “hubs”) coexists with a higher number of scarcely connected nodes [18]. From a dynamical point of view, these networks grow over the time, due to the attachment of new nodes preferentially to the hubs: the more one node is connected, the higher is the probability that it attracts a new node (preferential attachment) [19]. Thus, the probability *p*_*i*_ that the new node is connected to node *i* is:

$$ \mathrm{pi}=\mathrm{ki}\sum \mathrm{jkj} $$

where *k*_*i*_ is the degree of node *i* and the sum is made over all pre-existing nodes *j* [19, 20]. Finally, the node degree of nodes within the network follows a power-law distribution, i.e. the probability that a node has *k* links follows *P*(*k*) ~ *k* ^− γ^, where the degree exponent γ usually ranges between 2 and 3. Differently from other models of scale free networks such as the hierarchical networks, in BA networks the clustering coefficient, *C*(*k*), is independent of *k* and the average path length follows *l* ~ log log N [20, 21].

In this context, we looked for the most connected nodes, to identify biologically relevant molecules. The analysis of hubs offers very intriguing information and very thought-provoking data. Indeed, together with molecules well known as ubiquitous cell components (as it is the case of ATP) or as modulators of sperm physiology (see for instance Ca^2+^) we found several unsuspected nodes. They represent molecules specifically involved in function that are thought to be not present or not active in sperm cells, such as control of cell cycle, proteins synthesis, nuclear trafficking, and immune response. In all these cases, it will be very important to explore these functions in spermatozoa to verify the real biological meaning of this result. Anyway, this study, with other proteomic studies (see for reference [7]), could potentially open new perspectives on the study of sperm biology.

To date not all the researchers agree in definition of hubs and a univocal opinion on their topological and functional proprieties does not exists [22]. Anyway, our data seem to suggest that in sperm Interactome network it will be possible to differentiate hubs involved in control of a wide variety of functions and hubs devoted in controlling a specific function. In keeping with Han et al. [23] we analysed the clustering coefficient values of the most connected nodes within the network (the hubs) carrying out a kernel density estimation. As a result, we identified different subpopulations of nodes (see Additional file 3). The bigger one is characterised by very low values of this parameter, ranging from 0 to about 0.17, while the other one is smaller and its cluster coefficient is significantly higher, from 0.83 to 0.89. It is possible to speculate that the different clusterization of hubs could be related to a different functional meaning of the corresponding molecule in sperm physiology. This difference seems to be in agreement with the hypothesis of Han and colleagues, which defined two different types of hubs “‘party’ hubs, which interact with most of their partners simultaneously [and that are characterized by lower values of clustering coefficient], and ‘date’ hubs, which bind their different partners at different times or locations [and that display higher values of clustering coefficient]” [23].

For instance, here, the most important (in term of node degree) party hub is ATP, which is obviously involved in a myriad of chemical reactions in different times and subcellular locations, thus behaving as general hub. As energy substrate, in male germ cells ATP is produced by glycolysis and mitochondrial oxidative phosphorylation, the two major metabolic pathways expressed in spermatozoa that are localized in distinct cellular sub-compartments: glycolysis occurs mainly in the fibrous sheath of the flagellum where glycolytic enzymes are tightly anchored, while oxidative phosphorylation occurs in mitochondria, in the sperm midpiece [24]. The preferential use of one pathway is highly species specific [25].

The second subpopulation (date hubs) is characterized by a scarce clusterization and by the presence of members of the interferon family. As it is known in somatic cells, interferon is involved in immune response, being released in presence of pathogens such as viruses, bacteria, and parasites. In addition, interferon activates immune cells, such as natural killer cells and macrophages, and is able to increase host defences by up-regulating antigen presentation. Its function in spermatozoa has poorly studied, and there are only a few reports about its biological activity in mature male germ cells. During spermatogenesis, it has been supposed that INF could play a key role, as suggested by Hibi et al. [26] that found that the administration of α-IFN improved testicular spermatogenesis and increased the epididymal sperm concentration in the rat. Interestingly, they found also that progressive motility of the spermatozoa was unaffected by α-IFN treatment, while serum LH levels were decreased and serum testosterone an FSH levels were significantly increased. More recently, Satie et al. [27] compared a mouse strain transgenic for IFN_(Tg10) and a sister strain lacking the IFNAR1 subunit of IFNAR(Tg10-*Ifnar1*^_/_^), both strains expressing the transgene in the testis. The Tg10 mice, but not the double mutant Tg10-*Ifnar1*^_/_^, showed altered spermatogenesis. The most important IFNAR-dependent histological alteration was a higher apoptosis index in all germ cell categories that occurred 3 weeks after the onset of IFN production, at postnatal day 20. In addition, the Authors found that several known interferon-stimulated genes were up-regulated in Tg10 Sertoli cells and in prepachytene germ cells. At day 60, Tg10 males were sterile and Sertoli cells showed increased concentration of anti-Mullerian hormone and decreased production of inhibin B. Based on these findings, they concluded that type I interferon signalling could be involved in etiopathogenesis of male idiopathic infertilities by affecting the interplay between germ cells and Sertoli cells.

The distinction between date and party hubs could be important for designing drugs or diagnostic tools in clinical andrology [28].

A second class of nodes that exert a strong control is that of bottlenecks, mainly involved in control of information flow within the network. As for hubs, several molecules known to be involved in sperm physiology represent the bottlenecks (see Additional files 1 and 2). Interestingly, if compared to hubs, in bottlenecks there are less represented proteins (28 vs. 59, respectively) and more represented reactions (81 vs. 9, respectively), thus indicating a stronger relevance of control on information flow (see above, the results of KO mice analysis).

To assess the reliability of our theoretical data, we evaluated the results of the analysis of reproductive phenotypes of KO mice. In other words, we compared the data regarding HSIN3.0_MC hubs and bottlenecks after removal of correspondent genes and their effects on fertility. It is very interesting that we found that the percentage of the frequency of fertility phenotypes in hubs and in non-hubs nodes did not differ significantly (*p* = 0.21991) (see Table 3), while the deletion of genes codifying for the bottleneck protein in 100% of cases caused infertility.

This finding lead us to conclude that in our network the bottlenecks play a more important role than the hubs. The reason of this different biological function could be found in the specific topology of the network. Indeed HSIN3.0_MC is constituted by direct links (it is the union of several pathways) so it could be considered a regulatory network. As elegantly demonstrated by Yu et al. [29] in that case the bottleneck are more essential than hubs, while in interaction networks the hub are the main controller of networks.

Obviously, this experiment has important limitations due to the small number of bottlenecks, to the specie-specific differences, and to the impossibility to test non-protein nodes. Anyway, in our opinion, this finding could have important implications because it confirms the difference in term of function between regulatory and interaction networks and, from a more general point of view, it allows to explore a complex biological function by using an in silico (network)-in vivo (KO mice) by controlling their complexity [30].

**Fig. 3:**
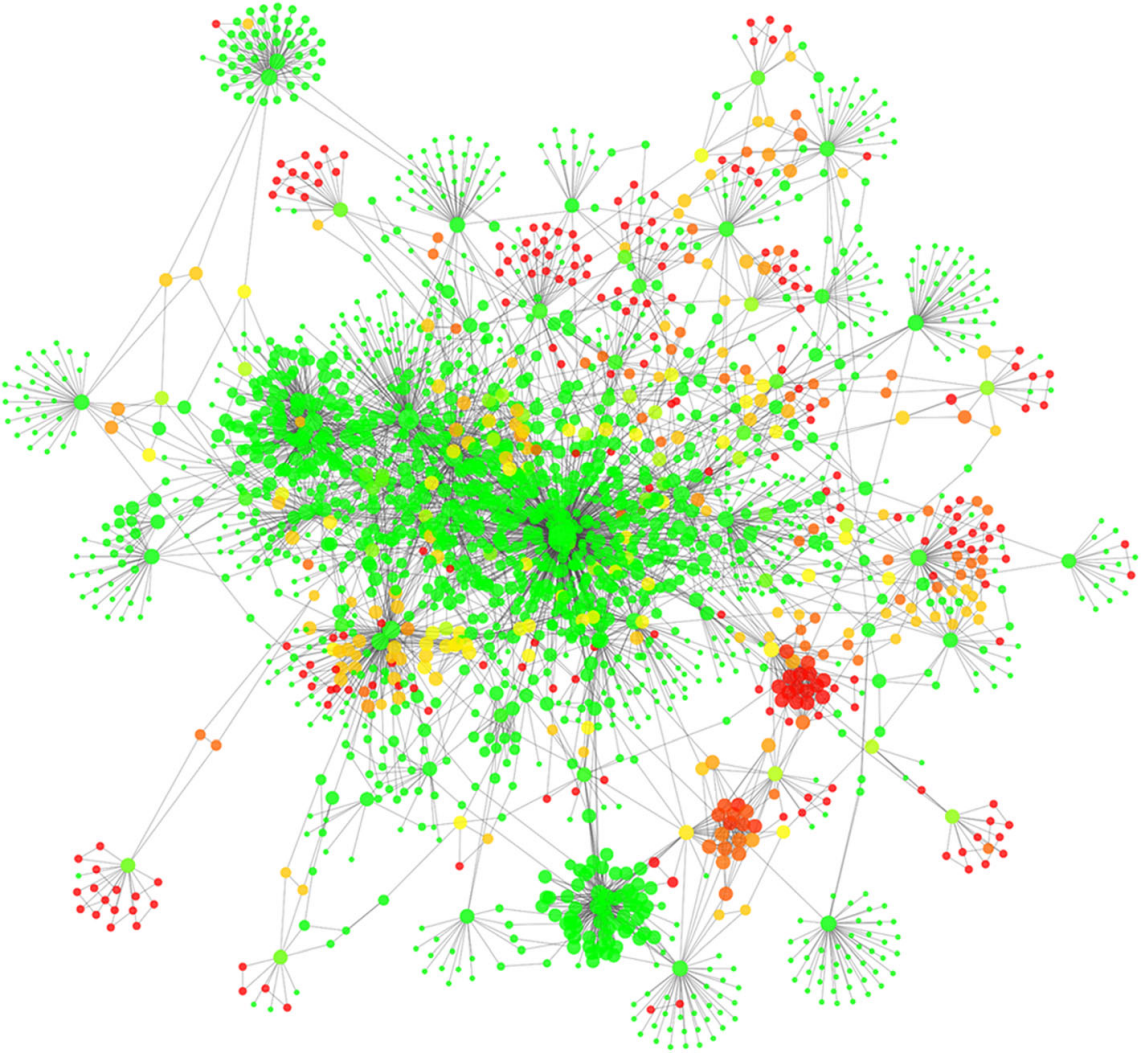
HSICN. The networks was spatially represented using the Cytoscape Prefuse Force Directed Layout. This program "is based on a" force-directed "paradigm. Network nodes are treated like physical objects that repel each other, such as electrons. The connections between nodes are treated like metal springs attached to the pair of nodes. These springs repel or attract their end points according to functional in force function. The layout algorithm sets the positions of the nodes in a way that minimizes the sum of forces in the network "(Cytoscape 3.4.0 User Manual http://manual.cytoscape.org/en/stable/index.html)). The node diameter is directly proportional to the degree node and the node color gradient was dependent from the clustering coefficient, from red (higher) to green (lower)

Once identified the hubs and the bottlenecks in HSIN3.0_MC, using the function “intersection” of Advanced Network Merge (in Cytoscape Tools menu) we computed the intersection of the network of most hubs with that of bottleneck, both of them obtained with cytoHubba. By this way, we obtained the network (Human Sperm Interactome Control Network, HSICN, see Fig. 3) that represents the backbone of control mechanism involved in spermatozoa physiology. Its analysis highlighted the idea that a few of nodes act as controllers of whole the network, allowing inferring two important biological implications. As first, we demonstrated that it is possible to create a map of control mechanisms of male gametes function, with enormous potential applications in the study of sperm physiology and pathology. Then, in several cases, the controllers are molecules poorly or not at all studied in spermatozoa. This, in one hand, could explain the inability to perform an etiological diagnosis in a large number of patients (unexplained infertility of male origin) and, on the other hand, could suggest several new targets for new studies.

Still looking at mechanisms active in leading sperm physiology, the results of protein characteristics analysis carried out using Uniprot are very interesting. As search parameters, we used factors known or supposed to be involved in sperm physiology and biochemistry, with the purpose to define the regulatory nodes within the network. We found that the protein present in HSIN3.0_MC are regulated by metal binding (12.3%), calcium binding (2.12%), pH (1.29%), and temperature (0.32%) (see Additional files 4, 5, 6 and 7) (we reported the percentages indicated by Uniprot, that are underestimated).

### Metal binding and calcium binding

It is known that spermatozoa biochemical machinery is under the control of metallic ions. Here we found that Mg^2+^, Ca^2+^, Zn^2+^, and Fe^2+^ are hubs of HSIN3.0_MC with 41, 37, 21, and 14 links. This datum is very interesting in the light of the presence in oviduct of metals (either in physiological conditions or in response to neuro-endocrine signals), in fact all of them are enzyme cofactors, thus concur in modulating whole biochemical machinery expressed by spermatozoa. For instance, it is known that the concentrations of Ca^2+^ in mammalian oviductal fluid immediately before ovulation are under hormonal control, and are involved in modulating the homeostasis and the hyperactivation of sperm motility [31], as well as Ca^2+^ is a key element in sperm capacitation and acrosome reaction [13, 16]. Indeed, spermatozoa are classified as excitable cells, such as neurons, i.e. as cells whose biological function is intimately calcium-dependent.

Moreover, zinc has been studied in a wide variety of experiments on different mammalian and non-mammalian models. In human andrology it has been found that seminal plasma zinc concentration is negatively associated with male infertility [32] and it has been suggested that its beneficial activity could be due to its antioxidant proprieties [33–35]. In addition this ion is known to interact with several enzymes such as acrosin, thus modulating their function.

pH: is spermatozoa intracellular pH is an important controller of cell function. As it has been recently reviewed, it “is essential for spermatozoa function. Notably, several unique sperm ion transporters and enzymes whose elimination causes infertility are either pHi dependent or somehow related to pH_i_ regulation” [36]. In particular, the molecules that are involved in pH_i_ control are CatSper, a Ca^2+^ channel; Slo, a K^+^ channel; the sperm-specific Na^+^/H^+^ exchanger and the soluble adenylyl cyclase, and are active in fundamental sperm functions such as motility, capacitation, and acrosome reaction.

### Temperature

The spermatozoa during their journey within the female genital tract experience site-specific differences in environmental temperature that originate a temperature gradient. To date, data concerning the temperature in human fallopian tube are not available; while in mammalian models (particularly in rabbit and sow) it has been found that the fertilization site in the oviduct is 1–2 °C warmer than the sperm reservoir [37]. In rabbit, it has been shown this temperature difference is time dependent and that it increases from 0.8 ± 0.2 °C before ovulation to 1.6 ± 0.1 °C after ovulation [38].

The mechanisms involved in this phenomenon are not yet completely understood, but three different determinants have been proposed:

1. The hormone-dependent release of a macromolecule, the acid mucus glycoprotein, that undergoes extensive endothermic hydration [39];
2. The counter-current heat exchange of blood: the cold blood from the ovarian vein cools the blood that directed to the oviduct (as it happens in testicular pampiniform plexus) [40];
3. The change in the source of blood supply: the warmer ovarian artery before ovulation and the cooler uterine artery after the ovulation [41].

Interestingly, to date, the specific signalling pathways involved in spermatozoa thermotaxis are unknown, while it has been understood that this prove is capacitation-dependent: only capacitated spermatozoa are thermotactically responsive [37].

## Conclusions

In conclusion, the network topology could explain important biological characteristics of the sperm cells biology. For instance, it confers to these cells a high robustness against random damages. In fact the relatively low number of nodes (in our model we estimate that the hub are 196 on 7758 nodes) that exert a strong control on network limits the probability that a random damage could have important consequences on the network itself (the probability is about 2.5%) [20]. Reasonably this specific behaviour could offer an important evolutionary advantage in terms of adaptation [42]. In addition, the existence of a non-democratic structure of control systems allows the cells to limit the energetic expenses: they could maintain the homeostasis controlling a small number (10^1^–10^2^) instead a myriad (10^3^–10^4^) of molecules, with an evident saving of time and energy [14]. Without considering that an increase in the number of molecules to be controlled will correspond to a non-linear increase in interactions, with a further relevant amount of energy consumption.

In addition, these networks are characterised by an ultra-small world topology and by a scarce clusterization. It means that the information will spread within the networks in a very low number of steps. In mean, all the messages starting from one node in HSIN3.0_MC will reach all the others 7757 nodes in 7.4 steps. This assures the maximal fastness and the minimal loss of messages integrity without loops or feed-backs, thus maximizing the performance of the system [14]. Finally, it allows to identify the nodes acting a higher degree of control on the network (hubs and bottlenecks) and to study their topology and their biological proprieties. Interestingly, the data from KO mice model seem to support the biological relevance of our findings and suggest a more important role of bottlenecks than hubs in our network.

In our opinion, these results could have important implication for the study of sperm biology and physiopathology, even more in light of the high percentage of cases of unexplained infertility of male origin [43].

## Methods

### Data collection and networks construction and analysis

To realize HISN we used the data concerning the pathways expressed in human mature spermatozoa found in recent (< 5 years) peer-reviewed literature on PubMed database, referred to the sperm phisiology. Once identified the pathways (See Table 4 for the list), in keeping with Bernabò et al. [17, 44], we downloaded them from Reactome (http://www.reactome.org/), which is a free, open-source, curated and peer-reviewed pathway database, whose aim is to offer bioinformatics tools for the visualization, interpretation and analysis of biochemical pathways involved in relevant biological events (see Table 4).

**Table 4:** pathways downloaded from Reactome, to realize HSIN3.0

Pathway	Pathway ID (Reactome)
Apoptosis	R-HSA-109581
Signalling to RAS	R-HSA-167044
Signaling by Wnt	R-HSA-195721
Signaling by EGFR	R-HSA-177929
Cell Cycle, Mitotic	R-HSA-69278
Regulation of mitotic cell cycle	R-HSA-453276
Cell Cycle Checkpoints	R-HSA-69620
Axon guidance	R-HSA-422475
Regulation of insulin secretion	R-HSA-422356
Signaling by Insulin receptor	R-HSA-74752
Protein folding	R-HSA-391251
Translation	R-HSA-72766
tRNA Aminoacylation	R-HSA-379724
L1CAM interactions	R-HSA-373760
Metabolism of amino acids and derivatives	R-HSA-71291
Triglyceride Biosynthesis	R-HSA-75109
Metabolism of nucleotides	R-HSA-15869
Gluconeogenesis	R-HSA-70263
Glycolysis	R-HSA-70171
Hexose transport	R-HSA-189200
Hemostasis	R-HSA-109582
Membrane Trafficking	R-HSA-199991
Nucleosome assembly	R-HSA-774815
Post-translational protein modification	R-HSA-597592
ABC transporters in lipid homeostasis	R-HSA-1369062
Signaling by Interleukins	R-HSA-449147
Cholesterol biosynthesis	R-HSA-191273
Metabolism of vitamins and cofactors	R-HSA-196854
Mitochondrial fatty acid beta-oxidation of unsaturated fatty acids	R-HSA-77288
Mitochondrial fatty acid beta-oxidation of saturated fatty acids	R-HSA-77286
Peroxisomal lipid metabolism	R-HSA-390918
Formation of ATP by chemiosmotic coupling	R-HSA-163210

The obtained network was called Human Sperm Interactome 1.0. To aggregate the same molecules with different ID an ad-hoc Python script has been realized to harmonize molecule IDs and their aliases.

After this step, we obtained HSIN 2.0. To complete the network with all the possible interaction among the proteins that compose it, we used Search Tool for the Retrieval of Interacting Genes/Proteins, STRING (http://string-db.org/). It is a database of known and predicted protein interactions, which includes direct (physical) and indirect (functional) associations (Franceschini et al., 2013). The interaction provided by the STRING search were filtered based on experimentally determined interaction, database annotated, automated text mining, the species (*Homo sapiens*), and confidence score > 900. After this enrichment step, we obtained HSIN 3.0.

The different networks have been realized and analyzed using Cytoscape 3.4.0 (http://www.cytoscape.org) and its plugins Netework Analyzer and CytoHubba. To assess the networks topology, we automatically measured the parameters listed in Table 5, considering the networks as directed.

**Table 5:** Topological parameters assessed in this study

Parameter	Definition
Connected Components	It is the number of networks in which any two vertices are connected to each other by links, and which is connected to no additional vertices in the network.
Number of nodes	It is the total number of molecules involved.
Number of edges	It is the total number of interactions found.
Clustering coefficient	It is calculated as *C*I = 2*n*I/*k*I(*k*I–1), where *n*I is the number of links connecting the *k*I neighbors of node I to each other. It is a measure of how the nodes tend to form clusters.
Network diameter	It is the longest of all the calculated shortest paths in a network.
Shortest paths	The length of the shortest path between two nodes *n* and *m* is *L*(*n*,*m*). The **s**hortest path length distribution gives the number of node pairs (*n*,*m*) with *L*(*n*,*m*) = *k* for *k = 1,2,…*
Characteristic path length	It is the expected distance between two connected nodes.
Averaged number of neighbors	It is the mean number of connections of each node.
Node degree	It is the number of interaction of each node.
Node degree distribution	It represents the probability that a selected nodes has *k* links.
γ	Exponent of node degree equation.
R^2^	Coefficient of determination of node degree vs. number of nodes, on logarithmized data.

### Identification of HSIN 3.0 hubs

In keeping with Bernabò et al. [14, 15], we identified the hubs within HSIN 3.0, as the nodes with a degree at least one standard deviation above the network mean.

### Identification of subpopulation in hubs, by kernel density estimation (KDE)

To identify eventual subpopulation of hubs based on their topological parameters, we assessed the probability density function of node degree, of clustering coefficient, and of betweenness centrality was estimated by kernel density estimation (KDE) [2]. It is a nonparametric analysis used to estimate the probability density function of a variable, thus allowing inferring if it constituted by subpopulations of data.

### Identification of bottleneck nodes within HSIN 3.0

We carried out the identification of bottlenecks within the network by using the Cytoscape plugin CytoHubba. It implements the following algorithm for bottleneck calculation: Let Ts be a shortest path tree rooted at node s. BN(v) = Σs∈V ps(v) where ps(v) = 1 if more than |V(Ts)|/ 4 paths from node s to other nodes in Ts meet at the vertex v; otherwise ps(v) = 0 (Chin CH et al., 2014).

### Identification of phenotypical effects of the deletion of gene relative to protein listed in HSIN3.0_MC in KO mice on male fertility

We studied the phenotypical effect on male fertility of removing the genes codifying for proteins present in HSIN 3.0 using Mammalian Phenotype Browser by Mouse Genome Informatics (MGI) (http://www.informatics.jax.org/), by selecting the MP: 0005389 (reproductive system phenotype).

### Classification of HSNI 3.0 proteins

The classification of HSNI 3.0 proteins was carried out using UniProt (http://www.uniprot.org/). In particular, we have filtered the data for:

Function:

- Calcium binding: denotes the position(s) of calcium binding region(s) within the protein;
- Metal binding: binding site for a metal ion
- pH dependence: the optimum pH for protein activity
- Temperature dependence: indicates the optimal temperature for enzyme activity and/or the variation of enzyme activity with temperature variation; the thermostability of the enzyme is also mentioned in this subsection, when known.

### Data analysis

All the statistical analysis have been carried out using Past3 (https://folk.uio.no/ohammer/past/).

## Additional files

Additional file 1: List of hubs. (XLSX 18 kb)

Additional file 2: List of bottleneck nodes. (XLSX 16 kb)

Additional file 3: Party and date hubs. (XLSX 10 kb)

Additional file 4: Nodes controlled by calcium binding. (XLSX 12 kb)

Additional file 5: Nodes controlled by temperature. (XLSX 8 kb)

Additional file 6: Nodes controlled by metal binding. (XLSX 39 kb)

Additional file 7: Nodes controlled by pH. (XLSX 11 kb)
